# Effects of Ball Milling Processes on the Microstructure and Rheological Properties of Microcrystalline Cellulose as a Sustainable Polymer Additive

**DOI:** 10.3390/ma11071057

**Published:** 2018-06-22

**Authors:** Yu Zheng, Zongqiang Fu, Dong Li, Min Wu

**Affiliations:** 1 College of Engineering, China Agricultural University, No. 17 QinghuaEast Road, Haidian District, Beijing 100083, China; zoo156penguin@yeah.net (Y.Z.); dongli@cau.edu.cn (D.L.); 2 School of Materials Science and Mechanical Engineering, Beijing Technology and Business University, No. 11 Fucheng Road, Haidian District, Beijing 100048, China; fzqxiaoqiang@163.com; 3 Engineering Research Center for Agricultural Equipment and Facilities, Ministry of Education, Beijing 100083, China

**Keywords:** microcrystalline cellulose, ball milling, physicochemical properties, starch, rheological properties

## Abstract

To investigate the effect of ball mill treatment of microcrystalline cellulose (MCC) on the rheological properties of MCC-polymer suspension, the structure and physicochemical characteristics of ground samples with different milling time and the rheological behaviors of MCC-starch suspensions were determined and comprehensively analyzed. During the ball milling process, MCC underwent a morphological transformation from rod-like to spherical shape under the combined effect of breakage and an agglomeration regime. The particle size and crystallinity index of MCC exhibited an exponential declining trend with ball milling time. All of the milled MCC samples presented a crystalline cellulose I_β_ structure whereas the MCC mechanically treated in a shorter time had better thermal stability. Rheological measurements of starch/MCC suspensions indicated that all the blended paste exhibited shear thinning behavior and ‘weak’ elastic gel-like viscoelastic properties over the whole investigated range owing to the formation of entangled network structure. The rheological behavior of starch/MCC pastes was strongly dependent on milling time and concentration of MCC samples. The increase in milling time of MCC samples resulted in the loss of rheological properties of starch/MCC pastes, where the size of the MCC playing a dominant role in affecting the properties of composite suspension. In addition, a possible network within starch/MCC suspensions was proposed.

## 1. Introduction

Cellulose is the most ubiquitous and abundant natural biopolymer on earth which is widely distributed in wood, plants (cotton, flax, hemp, jute, palm oil), marine animals (e.g., tunicates), algae, fungi, and bacteria. Microcrystalline cellulose (MCC) is a purified, partially depolymerized crystalline cellulose, and generally can be extracted from variety of cellulosic sources by acid hydrolysis, enzymatic hydrolysis, mechanical disintegration, oxidation technique, ionic liquid treatment, or combination of two or more of these methods [1,2]. Because of the mechanical strength of MCC, as well as it being chemically inactive, physiologically inert with attractive binding properties, low toxicity, renewability and biodegradability, MCC offered a significant opportunity for multiple uses in diverse fields, for example, in the pharmaceutical industry as a tablet excipient [3,4], in the food industry as a thickener and fat substitute [2], and also, as an additive in paper and composites applications [5,6]. 

Biopolymer blending is one of the most effective methods to create new biomaterials with desired properties, and for food and film applications biopolymers are MCC samples [2,7]. The properties of MCC-reinforced composites are dependent on the processing conditions, dispersion microstructure, and characteristics of MCC employed, such as degree of crystallinity, particle size and shape, mechanical properties, thermal stability, and porous structure [8,9,10]. 

For polyvinyl alcohol/cellulose nanoparticles (PVA/CNs) bionanocomposite solutions, the rheological behavior depends on the dispersion microstructure of CNs in PVA system [11]. By tuning the concentration of PVA/CN suspension, CNs morphology, CN and PVA weight ratio, PVA/CN suspension systems with desirable performances could be obtained. In addition, MCC crystals can effectively stabilize emulsions through proper dispersion and formation of a weak three-dimensional network, which is based on particle shape, size, and partial dual wettability defined by crystallinity [2]. It seems that the rheology of biopolymer systems depends on the dispersed microstructure of final products, and high performance and innovative biopolymers can be achieved by tailoring the characteristics of MCC. Therefore, investigations on the processing, structure, morphology, and rheological properties of MCC and MCC/polymer suspensions are of considerable scientific interest and practical significance for fully exploiting the capabilities of MCC [1]. 

Rheological properties of MCC-polymer composites have been investigated in previous studies, which is crucial for achieving a fundamental understanding of the processibility and structure-property relationship [8]. An increase in cellulose nanocrystal concentration enhanced the shear viscosity of film-forming solution owing to the perfectly compatible phases, improving the tensile properties of produced film [7]. On the contrary, the addition of MCC samples decreased the viscosity, storage modulus, and loss modulus of starch/cellulose pastes since amylose and MCC formed heterogeneous microstructures in the pastes [12]. Moreover, the decrease in aspect ratio of cellulose nanocrystals (CNCs) by acid hydrolysis resulted in a significant loss of rheological properties, and the aspect ratio of CNCs was theoretically predicted from the intrinsic viscosity using the Simha’s equation [1]. The composite suspensions fabricated by adding MCC to various polymers showed distinctive rheological properties due to the different interactions between MCC, polymer and other ingredients. Despite the successful application of MCC in promoting mechanical and rheological behaviors of composites in specific studies, MCC still faces certain challenges, such as the improvement of compatibility and dispersibility in hydrophobic matrices, and the control of particle size distribution (length, width, Aspect ratio) to design and process liquid suspensions, surface functionalization, and MCC-polymer blends [8]. In addition, chemical processing methods include complex procedures, and expensive reactants and processing steps [13]. 

A perfect and nonpolluted technique involves planetary ball milling that can initiate varying degrees of size reduction and reduce crystallinity without any chemicals, and can also modify the filler morphology which enhances its compatibility with polymer blends [13]. To our knowledge, although ball milling enables the purposeful execution of physical and chemical transformations in powdered materials, most previous studies rarely dealt with a definite structure-property relationship and seldom focused on the influence of the processing characteristics of fillers on the rheological behaviors of polymer systems [13,14]. It is necessary to scientifically understand the effects of structural changes and other variations on physicochemical characteristics produced by different ball milling time since milling of raw materials affects rheological properties of polymer composite suspensions. Starch (ST) is a carbohydrate polymer that is stored in roots and tubers of various plants and is currently receiving increasing attention owing to its multiple functional properties in food, pharmaceutical, and composite fields. In our study, commercial microcrystalline cellulose was subjected to ball milling process for the first time producing amorphous cellulose with different particle size, morphology and crystallinity. The structure and physicochemical properties of resulting ground powders were analyzed through a variety of technical methods and the rheological behaviors of starch/MCC suspensions were comprehensively compared. Hence, it is possible to establish the relationships between the characteristics of milled MCC samples and rheological behaviors of starch/MCC suspensions, providing a theoretical basis for the practical application of ball mill treatment in the development of sustainable MCC polymers.

## 2. Materials and Methods

### 2.1. Materials

Microcrystalline cellulose powder was kindly provided by Sigma-Aldrich Corp. (St. Louis, MO, USA). Maize starch was purchased from Beijing Quanfeng Starch Co. Ltd. (Beijing, China). Glycerol and Xylitol were purchased from Beijing Lanyi Chemical Co., Ltd. (Beijing, China) and used as received.

### 2.2. Grinding Method

Microcrystalline cellulose was grounded by a vacuum planetary ball mill machine (QM-1SP04, Nanjing University Instrument Plant, Nanjing, China) to obtain ultrafine powders. Ultrafine grinding was performed by mixing microcrystalline cellulose powders and ZrO2 balls (6–10 mm in diameter) in a stainless steel milling jar with a weight ratio of 1:10 and filling ratio of all substances 70% for 1 h, 2 h, 3 h, 4 h, 6 h, 8 h, 12 h, 16 h, respectively. The rotation speed of the disk and milling jar was set to 450 rpm with milling jar alternately rotated in the forward or reverse direction at intervals of 2 min. The samples obtained were denoted as MCC1.0 h, MCC2.0 h, MCC3.0 h, MCC4.0 h, MCC6.0 h, MCC8.0 h, MCC12.0 h, MCC16.0 h, respectively. Eventually, all powders prepared were stored in sealed bags for further use.

### 2.3. Particle Size Distribution Analysis (PSD)

The particle size distribution of microcrystalline cellulose powders were measured using a MASTERSIZER 3000 laser particle analyzer (Malvern Instruments Ltd., Malvern, UK) with measurement range of 0.01–3000 μm. On the basis of the particle size distribution curves obtained, values of D10, D50, and D90 could be calculated, where D10, D50, and D90 represent particle sizes of the 10th, 50th, and 90th percentiles of total volume.

### 2.4. Scanning Electron Microscopy Analysis (SEM)

The surface morphology analysis was performed using a S-3400N scanning electron microscopy (Hitachi Ltd., Tokyo, Japan) at an accelerating voltage of 15 kV. Microcrystalline cellulose powder was mounted on aluminum stubs with conductive carbon tape and platinum coated by ion sputter coater prior to imaging.

### 2.5. X-ray Diffraction Analysis (XRD)

The crystallinity of microcrystalline cellulose samples was evaluated using a X-ray diffractometer (Beijing General Analysis Co., Ltd., Beijing, China) equipped with a Cu Kα radiation source (λ = 1.54060 Å) at 36 kV and 20 mA. The scanning scope is 2θ = 5–40° at a rate of 1°/min in a step size of 0.02°. The crystallinity of microcrystalline cellulose samples was calculated using jade 5 software.

### 2.6. Fourier Transform Infrared Spectroscopy Analysis (FTIR-ATR)

Chemical structures of samples were characterized using a Bruker Fourier transform infrared spectrometer (Alpha, Bruker Optics Inc., Billerica, MA, USA) equipped with an attenuated total reflection (ATR) accessory in the range of 600–4000 cm^−1^ at a resolution of 4 cm^−1^ with an accumulation of 64 scans. 

### 2.7. Differential Scanning Calorimetry Analysis (DSC)

The melting and crystallization behavior of microcrystalline cellulose samples were investigated using a Thermal Analysis (TA) Instruments Q20 differential scanning calorimeter (corp.USA, New Castle, DE, USA) fitted with a refrigeration system and a purge gas as nitrogen. The instrument was temperature and enthalpy calibrated using indium standard procedures before measurements. An empty aluminum pan was used as a reference. The sample of 6 mg was equilibrated at 20 °C for 2 min and heated to 450 °C at a heating rate of 10 °C/min followed by cooling to 40 °C. The test data were recorded. Onset melting temperature and melting enthalpy (ΔH) of MCC samples were determined from the thermograms obtained using TA Instruments Universal Analysis software. 

### 2.8. Preparation of Polymer Composite Suspensions

Starch, MCC and plasticizers (glycerol and xylitol in a ratio of 1:1) were added to 95 mL of deionized water in the proportions shown in Table 1, followed by high-speed mechanical shearing for 10 min. In deionized water, the corresponding proportion of microcrystalline cellulose was first added, then starch and additives were added at a fixed weight ratio of 7:3, and the total weight of the dry matter was kept at 5 g. Afterwards, the suspensions were heated in a boiling water bath for 1 h to completely gelatinize the starch and then immediately cooled to 40 °C and equilibrated for 2 h to prevent the starch from regenerating before starting the rheological experiment.

### 2.9. Rheological Measurements

Rheological properties of starch/MCC suspensions were measured using a rheometer (AR 2000, TA Instrument, Inc., New Castle, DE, USA) with a standard aluminum parallel plate geometry (40 mm diameter) at 25 °C. Steady-state shear viscosity was performed in a shear rate range from 10 to 300 s^−1^. Strain sweep measurements were conducted in the strain range of 0.01% to 100% at a fixed frequency of 1 Hz to determine the linear viscoelastic region for each sample. Frequency sweep measurements were carried out over the angular frequency range of 0.1 to 10 Hz within the linear viscoelastic region. 

## 3. Results and Discussion

### 3.1. Particle Size Distribution

The particle size distribution of samples was shown in Table 2. A gradual shift to smaller particle sizes with increasing of milling time was observed, indicating that the microcrystalline cellulose powders were pulverized during mechanical grinding process [15]. After 1 h of ball milling, the most significant decrease in median particle sizes (D50) was observed, which was caused by the mechanical treatment of samples, leading to the fragmentation of microcrystalline cellulose particles and to the increase in the number of particles with the decrease in size. As the milling time prolonged, the rate of decline in D50 slowed down. It was worth noting that, in the ball milling 2–3 h process, the D10 decreased while the D90 increased, where D10 and D90 represented particle sizes of the 10th and 90th percentiles of total volume [14]. This was probably because the ball milling reduced heterogeneously the whole particle population and attacked a fraction of the larger particles to form smaller particles resulting in the decrease of D10 [14]. In addition, larger particles were generated by the aggregation of individual particles leading to an increase of D90, which indicated the simultaneous presence of breakage and agglomerative phenomena in this process [16]. After 3 h of grinding, there was a temporary platform appeared in the median particle size, which presumably suggested that the effect of large particles decomposition was weakened or neutralized by the accumulation of small particles, and hence preventing the particle size from decreasing. After 4 h, the D10, D50 and D90 of the microcrystalline cellulose powder continuously decreased, indicating that the milling process was dominated by the breakage phenomena. The samples after 12 h of mechanical treatment reached the ultimate particle size.

### 3.2. Surface Morphology

**Figure 1:**
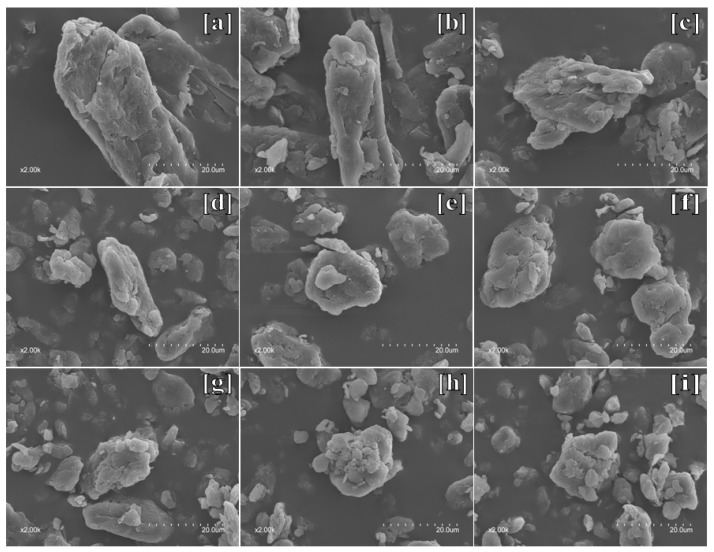
The morphology of the unmilled MCC sample and MCC samples with ball milling time of 1, 2, 3, 4, 6, 8, 12, 16 h, respectively, at 2000-fold magnification (**a**–**i**).

Figure 1 shows SEM micrograph of microcrystalline cellulose powders milling different time at 2000-fold magnification, which revealed the effect of mechanical milling process on the morphology of microcrystalline cellulose at micrometer level. The majority of the raw microcrystalline cellulose exhibited a distinct crystalline structure, which was typically a short rod-like shape with a rough surface (Figure 1a). After 1h of ball milling, the compact rigid structure of the cellulose crystallites transformed into a loosened needle-like structure exhibiting an intuitive reduction in size (Figure 1b). After 2 h of ball milling, the microcrystalline structure was crushed into small fragment, with a more pronounced decrease in length than in width, indicating that during this stage the main effect of the milling was the breaking of the cellulose crystallites along the direction perpendicular to the crystallite axis (Figure 1c) [17]. When milling for 3 h, it was clearly observed that several flaky particles were still remained in most of the ellipsoidal microparticles because of the insufficient ball milling treatment (Figure 1d) [18]. Subsequently, the pulverized particles were more uniformly dimensioned and exhibited a quasicircular shape after 4 h of grinding (Figure 1e). Furthermore, the surface of the particles was rough and porous along with fine particles attached to the surface of large particles, indicating the agglomerative phenomena in the powder [16]. The occurrence of these agglomerative phenomena was probably due to the high specific area and strong hydrogen bonds formed between the particles, which were difficult to be disrupt using a single mechanical disintegration method [11]. Ball milling for more than 6 h (Figure 1f), the clustered particle structure became more compact with smaller pores on the surface, resulting from energy collisions of the milling balls. After 8 h, various tightly clustered particles were further aggregated, whereas the size of the aggregates remained almost unchanged, which was consistent with the particle size results.

### 3.3. X-ray Diffraction

**Figure 2:**
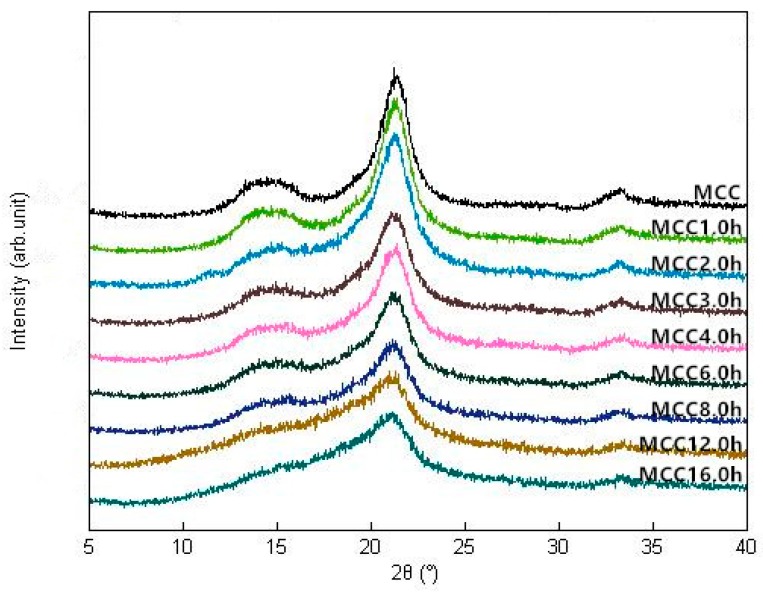
XRD patterns of MCC samples with different ball milling time.

As shown in Figure 2, the four characteristic diffraction peaks of cellulose I were observed at 2θ = 14.4°, 15.2°, 21.3° and 32.9° in all samples, corresponding to (110), (012), (200) and (040) crystallographic planes, respectively. These diffraction peaks of ground samples become wider and less resolved with increasing of the ball milling time compared to untreated sample, which showed a finer and more intense peak, indicating that the mechanical fragmentation did not disrupt all the crystal structure but had a significant influence on the crystallinity of treated samples [19]. The crystallinity index (CI) of microcrystalline cellulose was distinctly declined upon mechanical treatment from 56.34% of untreated sample to 25.15% of MCC16.0 h, suggesting that the ordered crystalline structure was partially damaged and the amorphization of cellulose progressed with milling time, leading to a continuous reduction in tensile strength of cellulose crystallites. The similar results were reported by Silva et al. (2012) [14] that ball milling disrupted the cellulose ordered structure by principally reducing crystallites thickness and slightly shortening crystallites. Karinkanta et al. (2014) [20] reported that the crystalline parts of cellulose crystallites were very strong and its fragmentation was mainly due to mechanical stressing in oscillatory ball milling rather than the particles-particle collisions during jet milling. After 12h, the CI remained essentially unchanged and then reached a platform at 16 h. CI was calculated as a function of ball milling time and their relationship could be characterized by *Y* = 23.07(*e*^−0.469X^) + 33.47(*e*^−0.01953X^) (*R*^2^ = 0.9952, *Y* represents the CI, *X* represents the milling time). Based on the energy consumption theory, two contrary mechanisms, that is, particle breakage and agglomeration of the so-created fragments, may lead to the achievement of grinding limit when no defects were generated in the crystalline lattice and the crystallite size was not further reduced [21,22]. 

### 3.4. Fourier Transform Infrared Spectroscopy (FTIR)

**Figure 3:**
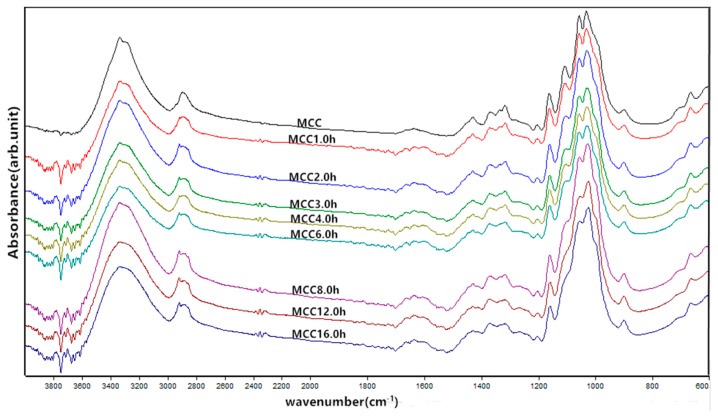
FTIR spectra of MCC samples with different ball milling time.

The effects of mechanical milling time on the chemical structure and composition of microcrystalline cellulose were elucidated by the FTIR analysis and the typical spectrum of the prepared MCC samples are shown in Figure 3. As milling time progressed, all MCC samples exhibited similar characteristic spectral band in two distinctive regions of the spectrum with one region presenting in 4000–2600 cm^−1^ and another “fingerprint” region specifying various stretching vibrations in 1800–400 cm^−1^. A strong band at 3335.4 cm^−1^ was corresponded to the stretching vibration of O-H groups and the band become less intense due to the reformation of more intermolecular hydrogen bonds between the free hydroxy groups or between free hydroxyl and water molecules after the breakage of the intermolecular and intramolecular hydrogen bonds in main chains of cellulose by the intensive milling impact [23]. A characteristic band around 2900 cm^−1^ was related to the stretching of asymmetric and symmetric C-H groups for the aliphatic moieties in polysaccharides. The band around 1637 cm^−1^ was attributed to the bending mode of the absorbed water and a contribution of some carboxylate groups, where the absorption peak separated into three connected small peaks after mechanical crushing and shown greater intensity because of the changes in the strength of intermolecular bonds and the strong interaction between cellulose and water [19,24]. Furthermore, the characteristic bands situated at 1428.3 cm^−1^ is indicative of the -CH_2_ bending, known as the crystallinity band, and an increase in its intensity was observed demonstrating a higher degree of crystallinity in MCC samples [25]. The bands located at 1367.4 cm^−1^ were ascribed to C-H bending or asymmetric C-H deformation and at 1315.2 cm^−1^ to CH_2_ wagging, while the band around 1160.8 cm^−1^ and 1056.5 cm^−1^ were assigned to the C-O-C stretching vibrations of skeletal glucose rings and pyranose, respectively [19,26,27]. The infrared band at 1106.5 cm^−1^ that associated to the glucose ring stretching completely disappeared after 8 h of grinding impact, whereas the absorption peak near 1030 cm^−1^ and 665.1 cm^−1^ was attributed to the stretching vibration of C-O and C-OH out of plane-bending mode, respectively [19]. In addition, the band at 897.7 cm^−1^ corresponded to the asymmetric out of plane ring stretching due to the β-linkage and the disordered form in cellulose [28]. It has been reported previously that the absorption around 3335.4, 2896.1, 1428.3, 1367.4, 897.7 cm^−1^ in all spectra indicated the characteristics of native cellulose I, and further the bands appeared at 3287.5 cm^−1^ for the -OH stretching and 665.1 cm^−1^ for out of plane bending were used to defined as cellulose I_β_ [19,29]. These results demonstrate that all MCC samples still remain in the cellulose I_β_ structure after ball milling process. No significant difference in the infrared spectrum was observed, suggesting that there was no change in the chemical structure of MCC after mechanical treatment.

### 3.5. Thermal Properties

**Figure 4:**
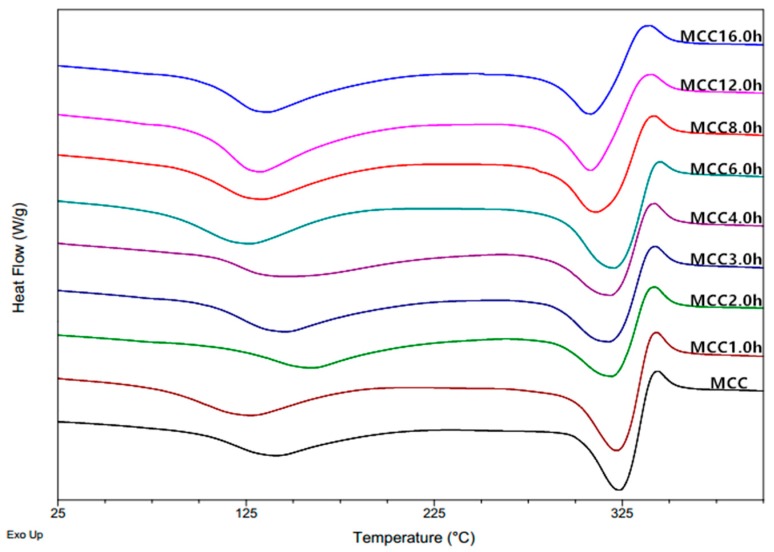
DSC curves of MCC samples with different ball milling time.

Differential Scanning Calorimetry (DSC) thermograms of microcrystalline cellulose samples are shown in Figure 4, giving two pronounced endothermic peak within the investigated temperature range. The first obvious and big endothermic peak of the DSC curves appearing from 30 °C to 150 °C is mainly related to the absorbed moisture evaporation [26]. It was found that the ∆H of water content of MCC powders increased from 145.2 J/g for the untreated sample to 153.7 J/g for MCC1.0 h sample, and further to 180.1 J/g, 191.9 J/g, 180.5 J/g, 184.1 J/g, 192.1 J/g, 236.8 J/g and 205.6 J/g for ball-milled samples at 2 h, 3 h, 4 h, 6 h, 8 h, 12 h, and 16 h, respectively. This was attributed to the changes in chemical and physical properties of microcrystalline cellulose during mechanically treatment, leading to differences in hygroscopicity and water holding capacity of the respective samples. For microcrystalline cellulose samples composed of amorphous and crystalline regions, the presence of loosely packed cellulose chains and prevalence of non-substituted hydroxyl groups in the amorphous regions lead to greater water absorption through the formation of hydrogen bonds than crystalline regions, and thus low crystallinity cellulose absorbs a high proportion of moisture after grinding process [30]. The last two peaks appeared above 200 °C in the DSC curves, which is associated with the cellulose pyrolysis, with the endothermic peak followed by the exothermic peak indicating the volatilization and subsequent charring process of the cellulose. It was obtained that melting onset temperature of products gradually decreased from 333.56 °C to 321.42 °C as a consequence of particle size reduction, meaning that the ball-milled crystallites with smaller size required less thermal energy in comparison with the untreated sample [26]. The thermal enthalpy of endothermic peaks also gradually decreased due to the increase of amorphous content through the mechanical treatment process, indicating that the amorphous cellulose is more susceptible to initial thermal degradation than crystalline cellulose [26]. Trache et al. (2014) [31] reported that the heat of decomposition and the crystalline index have the same trend because the occurrence of heterolytic thermolysis of glycosidic bonds along the ordered chains is difficult, which required high energy in the case of a high degree of molecular ordering. 

### 3.6. Rheological Characterization

**Figure 5:**
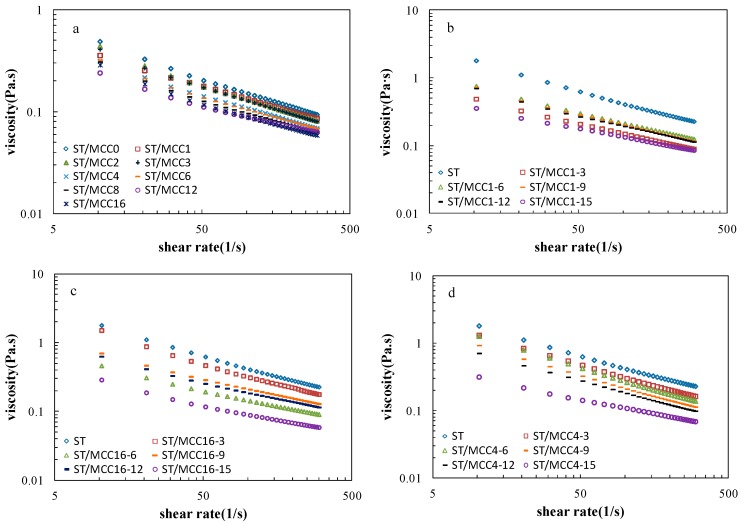
Shear viscosity versus shear rate of (**a**) ST/MCC suspensions with different ball milling times; (**b**) ST/MCC1.0 h suspensions; (**c**) ST/MCC4.0 h suspensions; and (**d**) ST/MCC16.0 h suspensions with concentrations of 15 wt % (purple circles), 12 wt % (black rectangles), 9 wt % (orange rectangles), 6 wt % (green triangles), and 3 wt % (red squares) of the total dry matter weight, and the control (blue diamonds) at 25 °C.

Steady-state viscosities as a function of the shear rate of ST/MCC blended pastes at various MCC milling time are shown in Figure 5a. The viscosity of all pastes similarly monotonically declined as the shear rate increased over the whole investigated shear rate ranges, exhibiting pseudoplasticity (shear-thinning) behavior [32]. In addition, MCC with a longer grinding time resulted in a decreased viscosity along the entire shear-thinning curve, displaying a weaker network structure. The possible reason is that the MCC particle dimensions (e.g., length and width) were reduced by the mechanical disruption and thus they could not offer strong resistance against the shear stress [11]. 

However, a portion of the mixed paste with specific MCC samples presented relatively higher viscosity over longer processing times, such as ST/MCC2.0 h, ST/MCC3.0 h, because of the decrease in crystallinity that led to an increase of hydroxyl groups on MCC surface to form more temporary hydrogen bonds prohibiting the viscosity reduction or even uplifted the viscosity. In this condition, it is concluded that the dimensions of MCC exerted a dominant effect on the viscosity of starch-based slurries.

Steady-state viscosities as a function of the shear rate of ST/MCC1.0 h, ST/MCC4.0 h, ST/MCC16.0 h blended pastes with MCC concentrations of 3, 6, 9, 12, and 15 wt % of the total dry matter weight are shown in Figure 5b–d, respectively. For all ST/MCC pastes, the typical shear thinning behavior was observed during shear flow and the viscosity gradually declined as MCC filling concentration increased due to the gelatinization inhibition in the presence of MCC molecules. It has been reported that MCC is an insoluble polysaccharide whose amorphous regions and the chain segments on the surface of crystalline region can absorb water and swell up at low filling concentration (lower than 6%), resulting in a decreased water content available for gelatinization in the hybrid system and thus a reduction of amylose leaching, granule swelling and enzymatic susceptibility with lower paste viscosity [12,33,34]. Furthermore, a heterogeneous system with an inhomogeneous distribution of amylose and MCC domains was formed in such paste, where the continuous phase was “diluted” by the MCC, impeding the network formation of dispersed amylose during gelatinization. On the contrary, a higher viscosity of the ST/MCC16.0 h paste was observed compared with the ST/MCC4.0 h and ST/MCC1.0 h paste due to the formation of intermolecular hydrogen bonds caused by the collisions of spherical MCC16.0 h particles improving the interfacial adhesion [35]. A similar result was reported by Zhou et al. (2016) [11]. When the MCC concentration increased by more than 6%, the correspondingly increased surface chain segment of swollen MCC granules have more chances to interact with amylose in the dispersion system, and these interactions lead to a reduced probability of starch helical formation, demonstrating a further drop in the viscosity as concentration increased [12]. Moreover, as the concentration of MCC increased, the reduced concentration of OH^-^ hydrophilic groups in the mixed system due to the decreased concentration of glycerol and xylitol is also one of the reasons for the reduction of the viscosity [36]. These results indicated that the viscosity of the ST/MCC-blended pastes was strongly dependent on shear rate, MCC concentration, and mechanical milling time.

**Figure 6:**
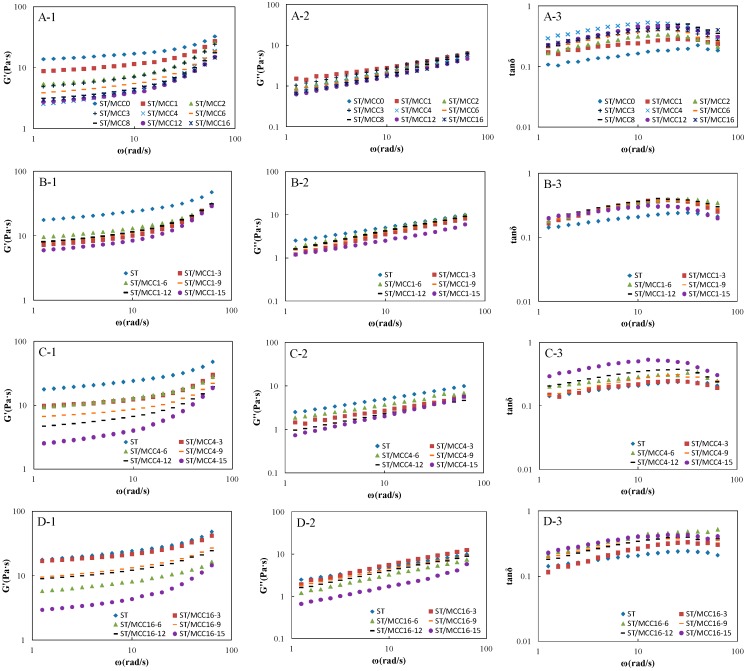
Dynamic modulus and loss tangent (G’, G’’ and tanδ) versus angular frequency (ω) of ST/MCC suspensions with different ball milling times (**A1**–**A3**); ST/MCC1.0 h suspensions (**B1**–**B3**); ST/MCC4.0 h suspensions (**C1**–**C3**); and ST/MCC16.0 h suspensions (**D1**–**D3**) with concentrations of 15 wt % (purple circles), 12 wt % (black rectangles), 9 wt % (orange rectangles), 6 wt % (green triangles), and 3 wt % (red squares) of the total dry matter weight, and the control (blue diamonds) at 25 °C.

Viscoelastic storage modulus (G′), loss modulus (G″) and tangent of the phase angle tan δ = G″/G′ of ST/MCC pastes as a function of angular frequency (ω) are shown in Figure 6. For all starch-based composite slurries, the G′ was basically higher than G″ (tanδ < 1) over the investigated frequency range with a certain frequency-dependency of either modulus, indicating a predominant ‘weak’ elastic gel-like behavior [1,12,37]. In addition, G′ and G″ decreased progressively with the increase of MCC concentration and ball milling time, indicating the dependence of viscoelastic behavior of mixed paste upon the concentration and ball milling time of MCC, which was strongly align with the the steady-flow rheological results. Donatella et al. reported that elastic behavior was a function of the number of effective chains participating in the formation of a network structure [38]. In general, there are mainly three possible interactions in starch-based composite pastes, physical entanglements between amylose, chemical interactions (e.g., hydrogen bond) between amylose, additives and MCC samples, and hydrogen bonds between MCC samples and immobilized water molecules, where the chemical interactions and hydrogen bonds were very weak but the physical entanglements were quite strong. Therefore, the chemical interactions between amylose, additives and MCC samples can be easily produced by adding MCC at different milling times or increasing the concentration of MCC, hindering the formation of amylose entanglement network.

## 4. Conclusions

The environmentally friendly and cost-competitive ball milling process reduced the particle size of microcrystalline cellulose in the micrometer range and destroyed the crystal structure of samples resulting in a decrease in crystallinity. In the process of ball milling, microcrystalline cellulose was transformed from rigid rod-like particles into ellipsoid loose clusters of particles and then into spherical compact aggregates under the combined effect of breakage and agglomeration regime. FTIR analysis demonstrated there was no change in the molecular structure and chemical structure of MCC samples with milling time. The MCC prepared in a shorter time have better thermal stability. All starch/MCC paste exhibited shear thinning behavior and ‘weak’ elastic gel-like viscoelastic properties over the whole investigated range owing to the formation of entangled network structure. The increase in milling time of MCC samples lead to a decrease in the viscosity of blended pastes, possibly due to the reduction in the MCC particle size and crystallinity, where the particle size of MCC samples playing a dominant role in affecting the properties of composite suspensions. As the concentration of MCC samples increased, the formation of heterogeneous microstructures and the decreased additive concentration reduced the viscosity of the mixed paste. The storage modulus and loss modulus of paste decreased progressively with the increase of MCC concentration and ball milling time due to the interference effect of microcrystalline cellulose on the formation of entanglement network. In general, the rheological behavior of composite slurries showed a significant dependence on milling time and concentration of MCC samples.

## Figures and Tables

**Table 1:** The proportion of the formulations of ST/MCC samples *.

Sample	Starch (g)	MCC (g)	Glycerol (g)	Xylitol (g)	Water (g)
Control (0%)	3.50	0.00	0.75	0.75	95.00
ST/MCC (3%)	3.39	0.15	0.73	0.73	95.00
ST/MCC (6%)	3.30	0.30	0.70	0.70	95.00
ST/MCC (9%)	3.19	0.45	0.68	0.68	95.00
ST/MCC (12%)	3.08	0.60	0.66	0.66	95.00
ST/MCC (15%)	2.97	0.75	0.64	0.64	95.00

* MCC represents the untreated or ball-milled microcrystalline cellulose; 0%, 3%, 6%, 9%, 12%, 15% represents the weight ratio of microcrystalline cellulose to the total dry matter.

**Table 2:** Particle size distribution and crystallinity index (CI) of microcrystalline cellulose samples.

Sample	D_10_ (μm)	D_50_ (μm)	D_90_ (μm)	CI (%)
Untreated	8.39 ± 0.24 ^a^	23.43 ± 0.32 ^a^	54.50 ± 0.61 ^a^	56.29 ± 0.08 ^a^
MCC1.0 h	5.35 ± 0.02 ^b^	15.90 ± 0.10 ^b^	40.43 ± 0.78 ^c^	47.89 ± 0.06 ^b^
MCC2.0 h	4.36 ± 0.06 ^c^	13.63 ± 0.21 ^c^	40.40 ± 1.51 ^c^	41.12 ± 0.09 ^c^
MCC3.0 h	4.18 ± 0.01 ^d^	13.20 ± 0.17 ^d^	48.73 ± 6.55 ^b^	36.44 ± 0.09 ^d^
MCC4.0 h	4.24 ± 0.04 ^c,d^	13.20 ± 0.17 ^d^	40.80 ± 3.70 ^c^	34.81 ± 0.08 ^e^
MCC6.0 h	3.76 ± 0.08 ^e^	11.50 ± 0.20 ^e^	29.97 ± 0.72 ^d^	31.63 ± 0.01 ^f^
MCC8.0 h	3.51 ± 0.07 ^f^	10.53 ± 0.06 ^f^	27.77 ± 0.47 ^d,e^	29.75 ± 0.03 ^g^
MCC12.0 h	3.21 ± 0.03 ^g^	9.72 ± 0.03 ^g^	24.70 ± 0.35 ^e^	25.24 ± 0.07 ^h^
MCC16.0 h	3.17 ± 0.09 ^g^	9.43 ± 0.12 ^g^	24.17 ± 0.40 ^e^	25.17 ± 0.02 ^h^

Data are shown as mean ± the standard deviation. Values in the same column with different superscripts are significantly different (*p* < 0.05).
